# MYCN and HDAC5 transcriptionally repress *CD9* to trigger invasion and metastasis in neuroblastoma

**DOI:** 10.18632/oncotarget.11662

**Published:** 2016-08-27

**Authors:** Johannes Fabian, Desirée Opitz, Kristina Althoff, Marco Lodrini, Barbara Hero, Ruth Volland, Anneleen Beckers, Katleen de Preter, Anneleen Decock, Nitin Patil, Mohammed Abba, Annette Kopp-Schneider, Kathy Astrahantseff, Jasmin Wünschel, Sebastian Pfeil, Maria Ercu, Annette Künkele, Jamie Hu, Theresa Thole, Leonille Schweizer, Gunhild Mechtersheimer, Daniel Carter, Belamy B. Cheung, Odilia Popanda, Andreas von Deimling, Jan Koster, Rogier Versteeg, Manfred Schwab, Glenn M. Marshall, Frank Speleman, Ulrike Erb, Margot Zoeller, Heike Allgayer, Thorsten Simon, Matthias Fischer, Andreas E. Kulozik, Angelika Eggert, Olaf Witt, Johannes H. Schulte, Hedwig E. Deubzer

**Affiliations:** ^1^ Clinical Cooperation Unit Pediatric Oncology, German Cancer Research Center (DKFZ) and German Consortium for Translational Cancer Research (DKTK), INF, Heidel­berg, Germany; ^2^ Phenex Pharmaceuticals AG, Waldhofer Straße, Heidelberg, Germany; ^3^ Department of Pediatric Hematology and Oncology, University Children's Hospital Essen, Essen, Germany; ^4^ Department of Pediatric Hematology, Oncology and SCT, Charité - University Hospital Berlin, Campus Virchow-Klinikum, Berlin, Germany; ^5^ Department of Pediatric Hematology and Oncology, University of Cologne, Cologne, Ger­many; ^6^ Center for Medical Genetics Ghent, Ghent University, De Pintelaan, Ghent, Belgium; ^7^ Cancer Research Institute Ghent, De Pintelaan, Ghent, Belgium; ^8^ Department of Experimental Surgery, Medical Faculty Mannheim, University of Heidelberg, Centre for Biomedicine and Medical Technology, Mannheim, Germany; ^9^ Department of Biostatistics, German Cancer Research Center (DKFZ), INF, Heidelberg, Germany; ^10^ Yale University, New Haven, CT; ^11^ Department of Neuropathology, University of Heidelberg, INF, Heidelberg, Germany; ^12^ Department of Pathology, University of Heidelberg, INF, Heidelberg, Germany; ^13^ Children's Cancer Institute, UNSW, Randwick, NSW, Australia; ^14^ Division of Epigenomics and Cancer Risk Factors, DKFZ, INF, Heidelberg, Germany; ^15^ Clinical Cooperation Unit Neuropathology, DKFZ, INF, Heidelberg, Germany; ^16^ Department of Oncogenomics and Emma Children's Hospital, Academic Medical Center, University of Amster- dam, Meibergdreef, Amsterdam, the Netherlands; ^17^ Neuroblastoma Genetics, DKFZ, INF, Heidelberg, Germany; ^18^ Kids Cancer Centre, Sydney Children's Hospital Randwick, Randwick, NSW, Australia; ^19^ Experimental Surgery and Tumor Cell Biology, University of Heidelberg, INF, Heidelberg, Germany; ^20^ Center for Molecular Medicine Cologne, University of Cologne, Cologne, Germany; ^21^ Max Plank Institute for Metabolism Research, Cologne, Germany; ^22^ Department of Pediatric Hematology and Oncology, Heidelberg University, INF, Heidelberg, Germany; ^23^ Junior Neuroblastoma Research Group, Experimental and Clinical Research Center of the Max-Delbrück Center for Molecular Medicine in the Helmholtz Community and the Charité - University Medicine Berlin, Lindenberger Weg, Berlin, Germany

**Keywords:** antimetastatic therapy, chromatin modulation, histone deacetylases, grainyhead-like transcription factor family, tetraspanin family

## Abstract

The systemic and resistant nature of metastatic neuroblastoma renders it largely incurable with current multimodal treatment. Clinical progression stems mainly from the increasing burden of metastatic colonization. Therapeutically inhibiting the migration-invasion-metastasis cascade would be of great benefit, but the mechanisms driving this cycle are as yet poorly understood. In-depth transcriptome analyses and ChIP-qPCR identified the cell surface glycoprotein, CD9, as a major downstream player and direct target of the recently described *GRHL1* tumor suppressor. CD9 is known to block or facilitate cancer cell motility and metastasis dependent upon entity. High-level *CD9* expression in primary neuroblastomas correlated with patient survival and established markers for favorable disease. Low-level *CD9* expression was an independent risk factor for adverse outcome. MYCN and HDAC5 colocalized to the *CD9* promoter and repressed transcription. *CD9* expression diminished with progressive tumor development in the *TH-MYCN* transgenic mouse model for neuroblastoma, and *CD9* expression in neuroblastic tumors was far below that in ganglia from wildtype mice. Primary neuroblastomas lacking *MYCN* amplifications displayed differential *CD9* promoter methylation in methyl-CpG-binding domain sequencing analyses, and high-level methylation was associated with advanced stage disease, supporting epigenetic regulation. Inducing CD9 expression in a SH-EP cell model inhibited migration and invasion in Boyden chamber assays. Enforced CD9 expression in neuroblastoma cells transplanted onto chicken chorioallantoic membranes strongly reduced metastasis to embryonic bone marrow. Combined treatment of neuroblastoma cells with HDAC/DNA methyltransferase inhibitors synergistically induced CD9 expression despite hypoxic, metabolic or cytotoxic stress. Our results show CD9 is a critical and indirectly druggable suppressor of the invasion-metastasis cycle in neuroblastoma.

## INTRODUCTION

Neuroblastoma, an embryonic tumor of neuroectodermal origin, accounts for 11% of all cancer-related deaths in children [1]. Molecular aspects create the extreme heterogeneity of this disease, spanning spontaneous regression to rapid metastasizing progression [1, 2]. Treatment scenarios range between observation only and multimodal concepts including high-dose chemotherapy with autologous stem cell rescue, surgery, radiotherapy and immunotherapy [3]. Despite decades of considerable international efforts to improve outcome, long-term survival of high-risk disease remains as low as 20% [3]. Managing resistance to induction therapy, causing tumor progression and early death in ultrahigh-risk patients, and managing chemotherapy-resistant relapses, which can occur years after initial diagnosis, remain major obstacles. *MYCN* amplifications [4, 5], telomerase activation by genomic rearrangements [6, 7], *ATRX* loss-of-function mutations or deletions [8–10] and germline or somatically acquired activating *ALK* mutations [11–14] define patient subgroups with highly aggressive and frequently therapy-resistant neuroblastomas. A commonality among these aggressive subgroups is that tumors undergo an as yet poorly understood invasion-metastasis cascade, which may be druggable to prevent increasing metastatic colonization.

Tetraspanin proteins all have four membrane-spanning domains, and form clusters between themselves and a large variety of molecules, predominantly including integrins, proteases and gangliosides. Tetraspanins can become engaged in signal transduction by recruiting cytoplas- mic kinases to the glycolipid- and cholesterol-enriched membrane microdomains to which they are localized [15–17]. CD9 tetraspanin expression strongly varies among different organs, with the highest expression found in endocrine tissues and the gastrointestinal tract, and the lowest expression reported both in liver and gall bladder [18]. Here, we assessed the significance of CD9 expression in primary neuroblastomas using clinical and molecular tumor data, unraveled the upstream activating and repressive control including epigenetic events and analyzed the functional role of CD9 using targeted CD9 re-expression in cell and animal models of neuroblastoma.

## RESULTS

### GRHL1 is a transcriptional activator of *CD9*

**Figure 1:**
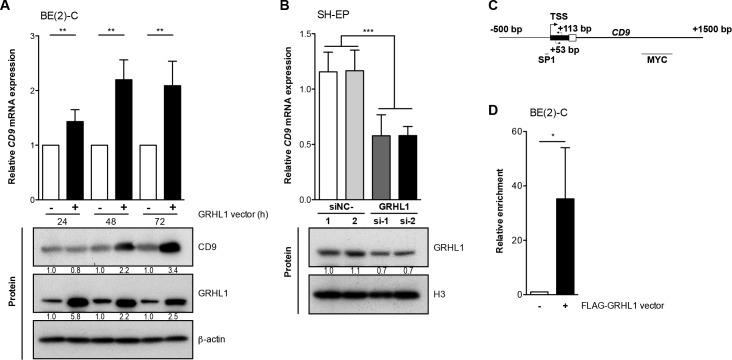
GRHL1 triggers CD9 expression **A.**, CD9 expression in BE(2)-C cells 24h-72h after *GRHL1* plasmid or empty-vector transfection on mRNA (upper panel; qRT-PCR; mean ± SD; *n* = 3) and protein level (lower panel; western blot*)*. **B.**, *CD9* expression in SH-EP cells 96h after GRHL1 knockdown with small interfering RNAs (si-1, si-2) (upper panel; qRT-PCR; mean fold-change over mock ± SD; *n* = 3). Controls were transfected with two different negative control siRNAs (siNC-1, siNC-2). GRHL1 knockdown efficacy was measured by western blot at 96h (lower panel) while CD9 protein expression in SH-EP was below detection. Histone H3 served as loading control. **C.**, schematic representation of the *CD9* promoter indicating the primer positions (arrows), the transcriptional start site (TSS) and the localization of previously described binding sites for SP1 and MYC. **D.**, the mean enrichment (± SD, *n* = 3) of *CD9* promoter DNA associated with GRHL1 is shown from ChIP-qPCR experiments. BE(2)-C cells from GRHL1 transfected cells were immunoprecipitated with antibodies against FLAG-GRHL1 or IgG, as negative control, and 0.1% input lysate served as normalization for comparing ChIPs (mean ± SD, *n* = 3). ^*^P < 0.05, ^**^P ≤ 0.01, ^***^P ≤ 0.001.

High-level GRHL1 expression favorably influences neuroblastoma biology at the molecular and phenotypic levels [19]. Time-resolved whole-genome expression analyses on BE(2)-C cells transfected with empty- or *GRHL1* vectors resulted in differential regulation over time of 170 genes, including *CD9* [19]. Increasing CD9 expression following enforced GRHL1 expression in BE(2)-C cells was confirmed both on the mRNA level by qRT-PCR and on the protein level by western blot analysis (Figure 1A). Transient knockdown of *GRHL1* in SH-EP cells by two different siRNAs to control for unspecific and off-target effects reduced *CD9* expression by approximately 50% (Figure 1B). We next conducted ChIP-qPCR using an antibody against the FLAG tag of FLAG-GRHL1 in overexpressing BE(2)-C cells to assess whether GRHL1 is recruited to the *CD9* promoter region (Figure 1C). GRHL1 was significantly enriched (Figure 1D) above the control (ChIP for IgG) in the *CD9* transcriptional start site distal to a previously described SP1 binding site [20] and proximal to a MYC binding site (http://genome.ucsc.edu). We conclude that GRHL1 activates *CD9* transcription in neuroblastoma cells.

### High-level *CD9* expression in neuroblastomas predicts favorable survival as an independent prognostic marker

**Figure 2:**
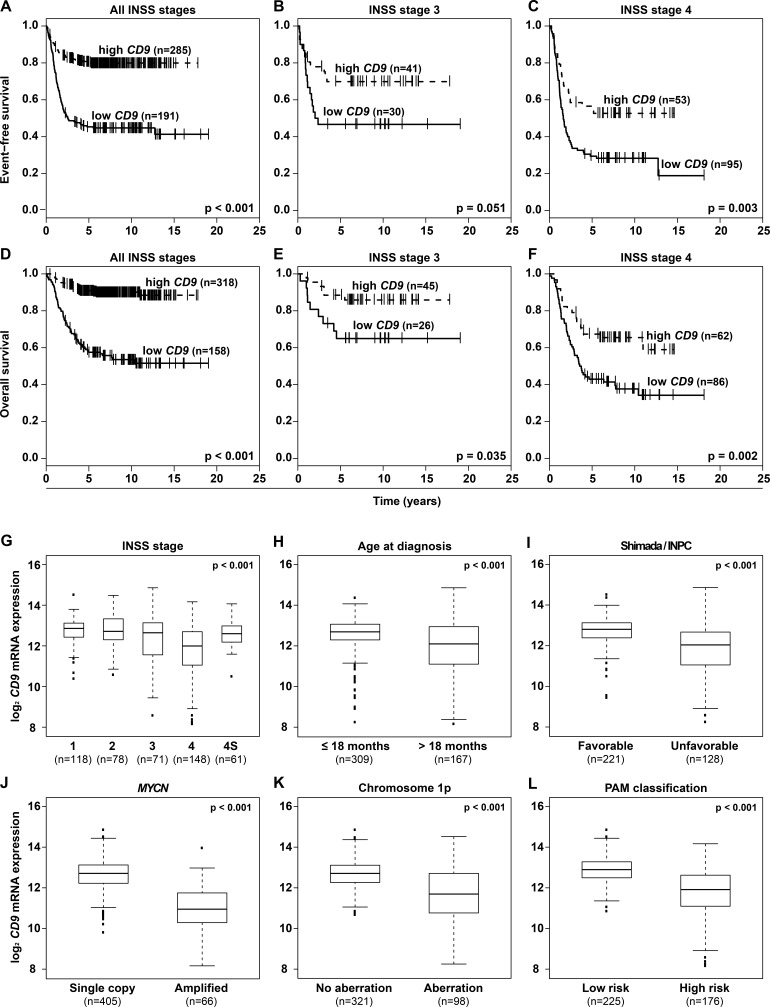
High-level *CD9* expression in primary neuroblastomas predicts favorable patient survival in a cohort of 476 tumors and correlates with favorable established clinical and molecular markers Kaplan-Meier analysis of event-free **A.-C.** and overall patient survival **D.-F.** irregardless of INSS stage **A.**, **D.** and in INSS stage 3 **B.**, **E.** and stage 4 patients **C.**, **F.** Box-plots compare *CD9* expression in tumors with varying stages according to INSS **G.**, in tumors from patients with age at diagnosis under or over 18 months **H.**, in tumors with favorable or unfavorable classification according to Shimada/INPC **I.**, in tumors either lacking or harboring *MYCN* amplifications **J.**, in tumors either lacking or harboring 1p aberrations **K.**, and in tumors classified either as low or high risk by a PAM classifier using microarray expression profiles **L.**

Having shown that *CD9* is downstream of GRHL1 in neuroblastoma cells, we assessed whether *CD9* is differentially expressed in primary tumors. We reanalyzed microarray expression data from a cohort of 476 neuroblastomas [21]. Kaplan-Meier analysis showed that high-level *CD9* expression in tumors, irregardless of International Neuroblastoma Staging System (INSS) stage or patient age, correlated both with favorable event-free and overall patient survival (Figure 2A-2F and Supplementary Figure S1A-S1D). These correlations were confirmed in microarray expression data from an independent cohort of 122 neuroblastomas [22] (Supplementary Figure S2A-S2D). High-level *CD9* expression in neuroblastomas from the 476-tumor cohort also significantly correlated with established clinical and molecular markers for favorable tumor biology, including INSS localized or 4S disease stages, age at diagnosis ≤ 18 months, favorable Shimada/International Neuroblastoma Pathology Classification (INPC) tumor histology, lack of *MYCN* amplifications or 1p aberrations and a low-risk tumor transcriptional profile previously defined by PAM analysis [23] (Figure 2G-2L). Correlations between elevated *CD9* tumor expression and all available clinical and molecular parameters for favorable tumor biology were confirmed in the 122-tumor cohort (Supplementary Figure S2E-S2H). These included localized INSS localized or 4S disease stages, age at diagnosis ≤ 18 months, lack of *MYCN* amplifications or 1p aberrations. Multivariate survival analysis was performed in the 476-tumor cohort to determine whether *CD9* expression provides additional predictive power over established clinical and molecular prognostic markers. Established risk factors were associated both with favorable event-free and overall survival in univariate survival analyses (Supplementary Table S3) and the prognostic value of *CD9* expression was independent of these established risk markers in multivariate analyses testing all factors together (event-free survival, *P* = 0.001; overall survival, *P* = 0.005; Supplementary Table S3). Taken together, high-level CD9 expression in primary neuroblastomas signals a favorable tumor biology and patient prognosis independent of established clinical and molecular risk factors.

### MYCN is a transcriptional repressor of *CD9*

**Figure 3:**
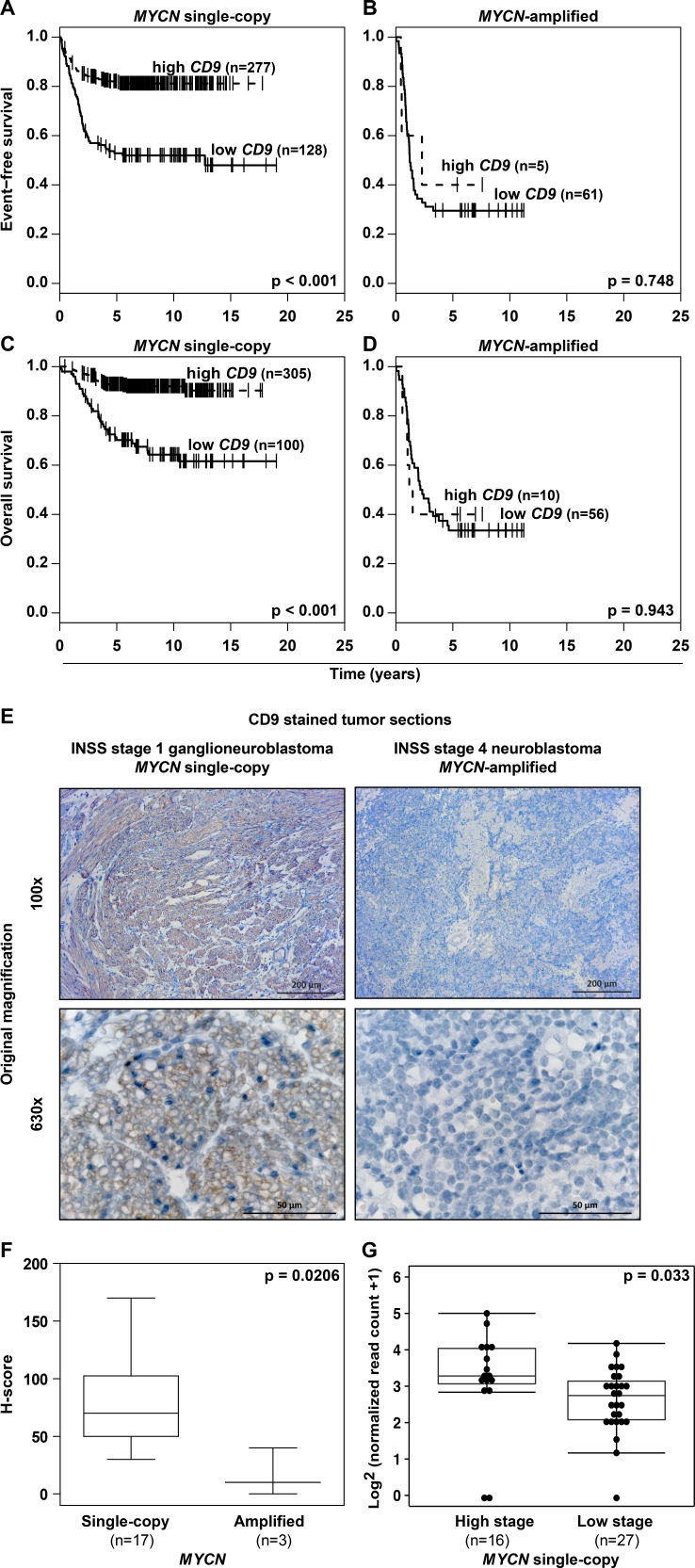
*MYCN* status and CD9 expression inversely correlate in primary neuroblastomas, and *CD9* promoter hypermethylation is associated with advanced stage disease in tumors lacking *MYCN* amplifications **A.-D.**, high-level *CD9* expression in *MYCN* single-copy but not in *MYCN*-amplified tumors predicts favorable patient survival in a cohort of 476 tumors. Kaplan-Meier analysis of event-free **A.-B.** and overall **C.-D.** patient survival. **E.-F.**, the differential pattern of *CD9* expression is translated to the protein level in neuroblastomas having the most divergent tumor biologies. CD9 immunohistochemical staining (brown) is exemplarily shown **E.** for a low-risk INSS stage 1 ganglioneuroblastoma lacking *MYCN* amplification and an INSS stage 4 poorly differentiated high-risk neuroblastoma harboring a *MYCN* amplification. CD9 expression was semi-quantitatively analyzed, and the data for all 20 tumors were presented as box-plots comparing the *MYCN* single-copy and *MYCN*-amplified tumor groups **F. G.**, Box-plots compare *CD9* promoter (chr12: 6308102-6310102; Human Genome Issue HG-19) normalized read counts of methyl-CpG-binding domain (MBD) sequencing data in high- (INSS stages 3 and 4) *versus* low stage *MYCN* single copy primary neuroblastomas (INSS stages 1, 2 and 4S).

**Figure 4:**
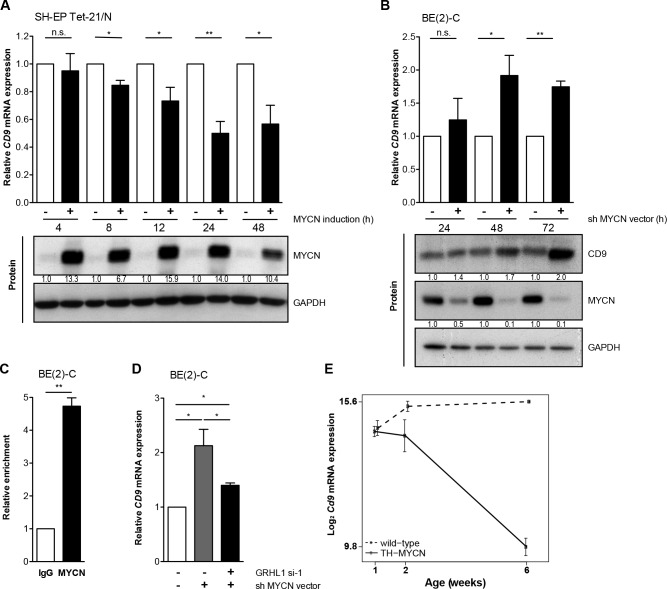
MYCN inhibits CD9 expression **A.-B.**, CD9 expression was assessed on the mRNA (qRT-PCR; mean ± SD, *n* = 3) and protein level (western blots) in the synthetic MYCN-inducible SH-EP Tet-21/N cell model with/without MYCN induction in time-course **A.** and in BE(2)-C at designated time points after MYCN depletion **B.** CD9 protein expression in SH-EP Tet-21/N was below detection. GAPDH served as loading control. **C.**, ChIP-qPCR showing an enrichment of *CD9* promoter DNA associated with MYCN. BE(2)-C lysates were immunoprecipitated with antibodies against MYCN or IgG, as negative control, and 0.1% of the input served for normalization (mean ± SD, *n* = 3). **D.**, *CD9* expression was assessed on the mRNA level (qRT-PCR; mean ± SD, *n* = 3) in BE(2)-C 96h after GRHL1 knockdown using a small interfering RNA (si-1) and 72h after MYCN depletion using a short hairpin RNA (shRNA) directed against *MYCN*. Controls were transfected with respective negative control siRNA and empty vector. **E.**, the expression of *Cd9* during neuroblastoma progression from tumor-prone ganglia to tumors in transgenic mice (*n* = 4; full line) and in comparison to *Cd9* expression in wild-type ganglia (*n* = 4; dashed line). Linear regression analysis; *P* = 4.7×10^−11^; delta slope = −1.1. ^*^P < 0.05, ^**^P ≤ 0.01.

A major factor influencing neuroblastoma biology is the *MYCN* status in the tumor, which inversely correlated with *CD9* expression in primary neuroblastomas (Figure 2J, Supplementary Figure S2G). Kaplan-Meier analyses comparing the predictive power of *CD9* expression in tumors lacking or harboring *MYCN* amplifications produced only a significant correlation between high CD9 expression and favorable event-free and overall survival in the patient cohort with tumors lacking *MYCN* amplifications (Figure 3A-3D), suggesting that MYCN may be upstream of *CD9*. To assess whether differential *CD9* expression in primary neuroblastomas with and without *MYCN* amplifications (Figure 2J, Supplementary Figure S2G) is mirrored at the protein level, we semiquantified CD9 expression immunohistochemically in 20 selected samples from tumors with the most divergent characteristics. Tumors lacking *MYCN* amplifications expressed CD9 in a variably strong manner, and expression was localized to the cell membrane, whereas no CD9 expression was detected in any of the *MYCN*-amplified tumors (Figure 3E-3F). We investigated *CD9* gene methylation status by reanalyzing existing methyl-CpG-binding domain sequencing data from 60 neuroblastomas [24, 25]. The *CD9* promoter was differentially methylated within the group of tumors lacking *MYCN* amplifications (*n* = 43), and high-level promoter methylation was associated with advanced stage disease as defined by INSS stages 3 and 4 (Figure 3G). These results confirm the inverse correlation between *MYCN* status and CD9 expression at the protein level and point towards a functional role of *CD9* promoter methylation in tumors lacking *MYCN* amplifications. We next used several different neuroblastoma cell and mouse models to investigate the nature of this relationship and the influence of MYCN on *CD9* expression. We analyzed changes in *CD9* expression occurring between 4h to 48h in the synthetic MYCN-inducible system, SH-EP Tet-21/N. Conditional MYCN expression downregulated *CD9* expression by approximately 15% after 4h and by 50% after 24h (Figure 4A). Endogenous MYCN expression was depleted in BE(2)-C cells by transient expression of a short hairpin RNA (shRNA) plasmid directed against *MYCN.* CD9 expression increased between 24h and 72h time-dependently, reaching a 2-fold increase in *CD9* mRNA expression and a 2.4-fold increase in CD9 protein expression (Figure 4B). Results from these two models suggest that MYCN suppresses CD9 expression. We performed ChiP-qPCR experiments to test whether MYCN directly associates with the *CD9* promoter. Not only was MYCN enriched in the *CD9* promoter (compare Figure 4C with Figure 1C), but this enrichment was confined to the region enriched for GRHL1 (Figure 1D). We have previously shown that MYCN is a transcriptional repressor of *GRHL1* [19]. To test whether GRHL1 is required for *CD9* induction mediated by MYCN depletion, we transiently knocked down both GRHL1 and MYCN in BE(2)-C. Transient GRHL1 knockdown diminished induction of the *CD9* transcript by MYCN depletion by 35%, indicating that GRHL1 contributes to *CD9* transcriptional activating when silencing MYCN (Figure 4D). We next used the TH-MYCN neuroblastoma progression model to analyze the temporal *CD9* expression pattern. *CD9* expression was strongly reduced during progressive development of neuroblastic tumors in TH-MYCN^+/+^ mice and remained far below *CD9* expression in ganglia from wildtype TH-MYCN^−/−^ mice (Figure 4E). Collectively, our data argue for a role of MYCN in the transcriptional repression of *CD9* in neuroblastoma cells.

### HDAC5 negatively regulates *CD9* and combined inhibition of HDAC5 and DNA MTases synergistically induces *CD9*

**Figure 5:**
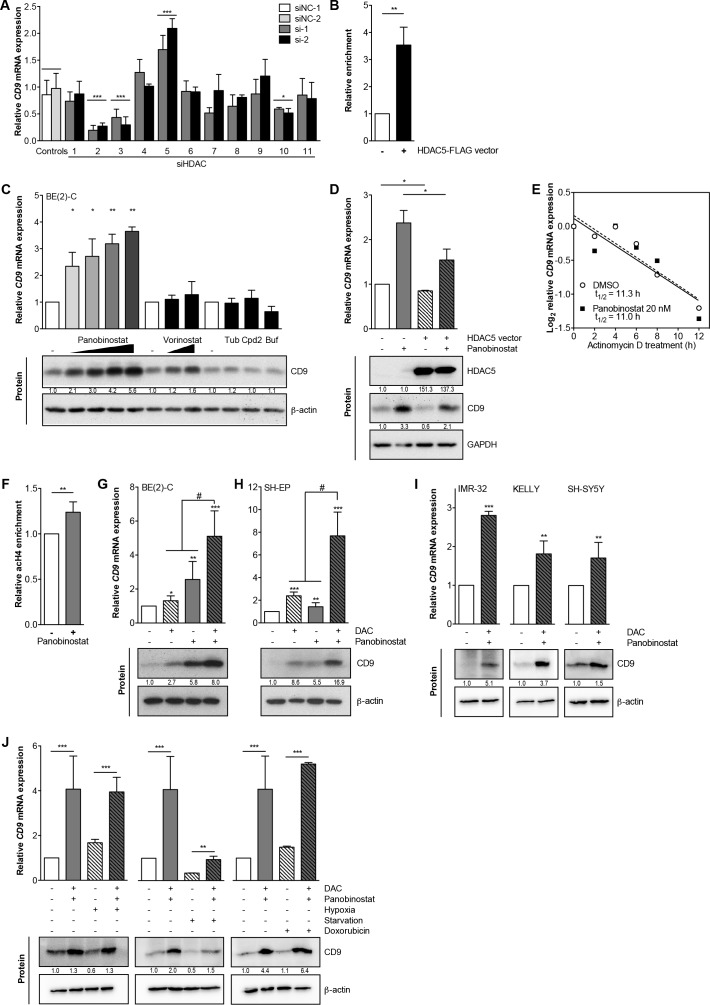
HDAC5 negatively regulates *CD9*, and combined DAC/HDAC inhibitor treatment synergistically triggers CD9 expression **A.**, RNAi targeting HDACS 1-11 singly in BE(2)-C cells with siRNAs (si-1, si-2). Control were transfected with two different negative control siRNAs (siNC-1, siNC-2). *CD9* expression was measured by qRT-PCR 96 hours after transfection and is represented as mean fold-change over mock control ± SD (*n* ≥ 2). **B.**, ChIP-qPCR (mean ± SD, *n* = 3) showing an enrichment of *CD9* promoter DNA associated with HDAC5. BE(2)-C lysates were immunoprecipitated with an antibody against HDAC5 or IgG as negative control. **C.**, CD9 expression (assessed by qRT-PCR and Western blotting) after treating BE(2)-C cells with panobinostat (5 nM, 10 nM, 15 nM, 20 nM), vorinostat (0.5 μM, 1 μM), 5 μM tubacin (Tub), 20 μM compound 2 (Cpd2), 30 μM bufexamac (Buf) or respective solvent controls for 48 hours (mean fold-change over control ± SD is shown, *n* ≥ 3). **D.**, CD9 expression (assessed by qRT-PCR and Western blotting) in BE(2)-C cells transfected with HDAC5 or empty vector for 72h and treated with 15 nM panobinostat or solvent control for 48h. GAPDH served as loading control. **E.**, *CD9* expression (assessed by qRT-PCR) in BE(2)-C cells treated up to 12 hours with 1 μg/ml Actinomycin D, 20 nM panobinostat or solvent control (*n* = 3). **F.**, the mean enrichment (±SD, *n* = 3) of *CD9* promoter DNA associated with pan-acetylated histone H4 after 5 hours of 20 nM panobinostat treatment is shown from ChIP-qPCR experiments. BE(2)-C lysates were immunoprecipitated with an antibody against pan-acetylated histone H4, and 0.1% input lysate was used as a loading control for comparing ChIPs. **G.-H.**, CD9 expression was assessed on the mRNA (qRT-PCR; mean ± SD, *n* = 3) and protein level (western blotting) in the neuroblastoma cell lines BE(2)-C **G.** and SH-EP **H.** 96 hours after treatment with 3 μM DAC, 10 nM panobinostat or combined 3 μM DAC/10 nM panobinostat treatment. β-actin served as loading control. **I.**, CD9 expression in the neuroblastoma cell lines IMR-32, Kelly and SH-SY5Y 48 hours after combined treatment with 3 μM DAC and 5 (IMR-32) - 10 nM panobinostat (Kelly, SH-SY5Y) or solvent controls. J, RNA and protein were isolated from BE(2)-C cells 48 hours after combined treatment with 3 μM DAC/10 nM panobinostat or solvent controls under normoxia (21% O_2_) *versus* hypoxia (3% O_2_), serum substitution (10% FCS) *versus* starvation (0.1% FCS) and normal condition *versus* genotoxic stress (0.1 μg/ml doxorubicin). CD9 expression was measured at the mRNA and protein levels using qRT-PCR (mean fold-change over solvent ± SD, *n* = 3) and western blotting. DAC, 5-aza-2′-deoxycytidine. ^*^P < 0.05; ^**^P ≤ 0.01; ^***^P ≤ 0.001; ^#^*P* = 0.0131 for BE(2)-C and *P* ≤ 0.0001 for SH-EP.

A model for transcriptional repression *via* MYCN involves histone deacetylase (HDAC) recruitment to promoter regions. To identify HDACs potentially involved in *CD9* transcriptional repression, we transiently knocked down each of the eleven HDACs (using two different siRNAs for each to control for unspecific and off-target effects, Supplementary Table S2) belonging to classes I, II a/b and IV in BE(2)-C cells, then assessed *CD9* expression. Only HDAC5 depletion induced *CD9* expression, which reached 1.7- to 2.3-fold of the control 96h after knockdown (Figure 5A). Depleting HDACs 1, 4, 6, 7, 8, 9 and 11 did not affect *CD9* expression, while depleting HDACs 2, 3 and 10 decreased *CD9* levels (Figure 5A), suggesting HDACs 2, 3 and 10 may counteract HDAC5 influence on *CD9* expression. To test whether HDAC5 is recruited to the *CD9* promoter site, we performed ChIP-qPCRs using anti-FLAG M2-conjugated agarose to capture immunocomplexes after HDAC5-FLAG enforced expression in BE(2)-C cells. HDAC5 was enriched by ∼3.5-fold at the *CD9* promoter (compare Figure 5B with Figure 1C), and localized to the region where GRHL1 was also enriched as a transcriptional activator and MYCN was enriched as a transcriptional repressor.

As an alternative approach to HDAC5 knockdown, we treated BE(2)-C cells with a variety of small molecule HDAC inhibitors, then assessed CD9 expression. CD9 induction by the clinically approved pan-HDAC inhibitor, panobinostat, was dose-dependent, and ranged between 2.3- and 3.8-fold on the mRNA level and between 2- and 5.3-fold on the protein level (Figure 5C). Another clinically approved HDAC-inhibitor, vorinostat, weakly inhibited HDAC5, and only induced *CD9* expression by 1.4-fold and CD9 protein levels by 2-fold (Figure 5C). CD9 mRNA or protein expression was not induced by the HDAC6-selective inhibitor, tubacin; the HDAC8-selective inhibitor, compound 2; or by bufexamac, which inhibits both HDAC6 and HDAC10 (Figure 5C). To test whether enforced HDAC5 expression could at least partially counteract CD9 induction by HDAC inhibitor treatment, BE(2)-C, Kelly and SH-EP cells were transiently transfected with a HDAC5 expression vector then treated with panobinostat. Enforced HDAC5 expression decreased the CD9 induction triggered by panobinostat up to 15% on both transcript and protein levels (Figure 5D, Supplementary Figure S3A-S3B), demonstrating that the effect of pan-HDAC inhibition could be partially outcompeted by HDAC5 overexpression. To differentiate between *de novo* transcript synthesis and increased transcript stability, we cotreated BE(2)-C cells with actinomycin D and panobinostat or solvent control for up to 12h. Blocking *de novo* RNA synthesis with actinomycin D resulted in equivalent *CD9* expression in cells treated with HDAC inhibitor and untreated control cells, confirming that control is enacted *via* transcriptional activation (Figure 5E). To examine epigenetic changes prior to *CD9* transcriptional activation, we performed chromatin immunoprecipitation (ChIP) in BE(2)-C cells treated for 5h with panobinostat or solvent control using an antibody against pan-acetylated histone H4, and looked for changes in the *CD9* promoter. We detected an increase in pan-acetylated histone H4 associated with the transcriptional start site of the *CD9* gene after HDAC inhibitor treatment, indicating that epigenetic changes preceded transcriptional activation (compare Figure 5F with Figure 1C). Taken together, *CD9* is epigenetically and transcriptionally repressed by the chromatin-modifying enzyme, HDAC5, in association with MYCN, and CD9 expression can be triggered in neuroblastoma cells by inhibition of HDAC5.

Next, we assessed whether co-treatment with a nucleoside inhibitor of DNA methylation, 5-Aza-2′-deoxycytidine (DAC), could enhance transcriptional activation. Combined treatment of BE(2)-C and SH-EP neuroblastoma cells with panobinostat and DAC synergistically induced CD9 up to 8-fold on the transcript and 6.5-fold on the protein levels (Figure 5G-5H). We also achieved similar results using the IMR-32, Kelly and SH-SY5Y neuroblastoma cell lines (Figure 5I). We next analyzed the effectiveness of CD9 induction in BE(2)-C cells by combined panobinostat/DAC treatment under conditions mimicking those in tumors such as hypoxia, starvation and stress induced by cytotoxic drugs such as doxorubicin. Starvation caused by culture for 72h in serum-free medium strongly reduced endogenous CD9 levels, while both hypoxia and genotoxic stress had no major effect on CD9 expression (Figure 5J). Drug treatment triggered CD9 expression by 4- to 5-fold compared to controls in all culture conditions (Figure 5J), suggesting transcription can still be robustly activated. Our data argue a role for HDAC5 in transcriptionally repressing *CD9* in neuroblastoma cells beyond the repression achieved by MYCN and DNA methyltransferases, and offers an avenue for pharmacological intervention.

### CD9 attenuates neuroblastoma cell migration, invasion and metastasis formation

**Figure 6:**
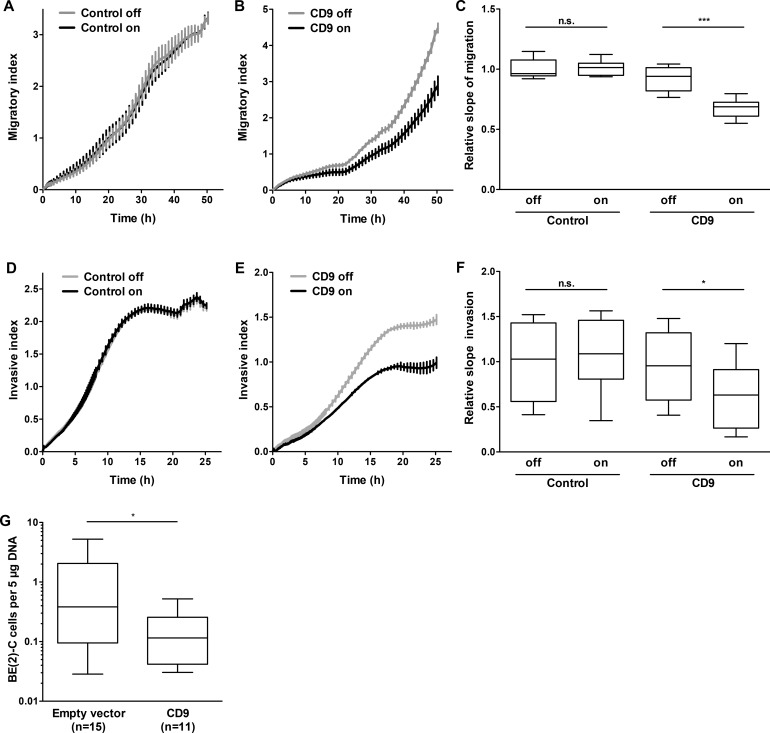
High-level CD9 expression inhibits migration and invasion of neuroblastoma cells in Boyden chambers and metastatic disease to the chicken embryo bone marrow The stable CD9-inducible SH-EP cell model and the respective stable control cells were used to determine the effect of CD9 expression on migration **A.-C.** and invasion **D.-F.** Migration/invasion was measured with the xCELLigence Real-Time Cell Analyzer (*n* = 3 in quadruplicate). **E.**, number of metastasizing BE(2)-C cells with and without enforced CD9 expression in the bone marrow chicken embryos. Cells were transplanted onto the chorioallantoic membranes of fertilized pathogen-free leghorn eggs (*n* ≥ 11 per study group). On day 17, the embryos were sacrificed, and the bone marrow was isolated. Human *ALU* sequences were amplified and quantified by qRT-PCR. n.s., not significant; ^*^P < 0.05; ^***^P ≤ 0.001.

**Figure 7:**
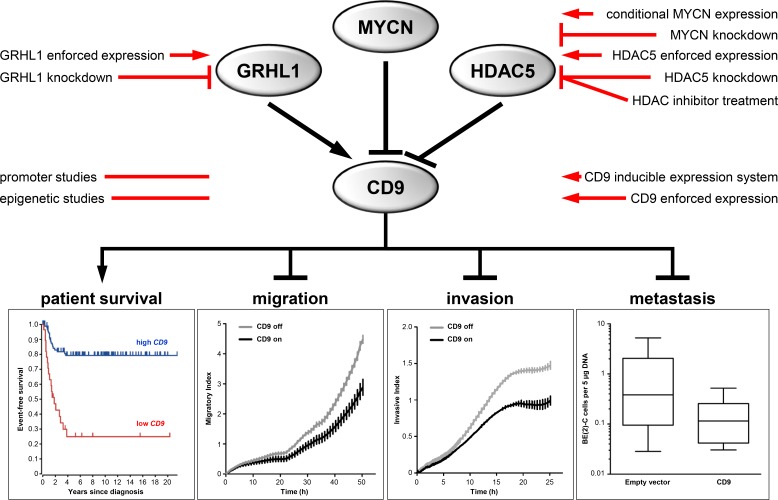
Schematic model summarizing the expression, regulation and function of CD9 in neuroblastoma and indicating the experimental strategies applied

Data obtained from primary tumors suggested that high-level *CD9* expression is associated with favorable tumor biology. To investigate whether high-level CD9 expression exerts its antitumorigenic effect *via* influencing proliferation and viability, SH-EP cells stably transfected with an inducible *CD9* construct or a negative control expression system (Supplementary Figure S4A-S4B) were analyzed in proliferation assays. CD9 induction did not significantly alter cell proliferation or viability in the SH-EP cell model (Supplementary Figure S5A-S5B). Similarly, transient enforced CD9 expression did not alter BE(2)-C cell proliferation or viability compared to control cells transfected with empty or LacZ vectors (Supplementary Figure S4C-S4D and Supplementary Figure S5C-S5D). Xenograft tumor take or growth rate was also not influenced by prior transient transfection with either *CD9* or LacZ control vector in BE(2)-C cells subcutaneously implanted into CB17-SCID mice (Supplementary Figure S5E). These experiments strongly indicate that CD9 exerts its antitumoral effects *via* other avenues than influencing tumor cell proliferation. To investigate whether migratory or invasive capacity was affected, we functionally analyzed the phenotypic consequences of inducing CD9 expression in the SH-EP cell model during Boyden chamber migration and invasion assays. Inducing CD9 expression reduced cell migration by 40%, while inducing the negative control expression system produced no difference in cell migration (Figure 6A-6C). Similarly, CD9 induction inhibited invasion capacity by 40%, while inducing the negative control expression system again had no effect (Figure 6D-6F). We next evaluated whether our *in vitro* findings were also applicable in the *in vivo* chicken embryo chorioallantoic membrane metastasis assay. The number of BE(2)-C cells that metastasized from the chorioallantoic membrane to the chicken embryonic bone marrow was strongly reduced by enforced CD9 expression compared with empty vector-transfected control cells (Figure 6G). SH-EP cells, which do not metastasize in the chicken embryo chorioallantoic membrane metastasis assay, were not investigated. Transient enforced expression was applied in BE(2)-C cells as inducible gene expression by tetracycline addition is technically not feasible in the chicken embryo chorioallantoic membrane metastasis assay. Taken together, our data supports a mode of action by CD9, in which high-level expression in neuroblastoma cells exerts an antitumoral effect by inhibiting several different steps in the invasion-metastasis cascade (schematic model shown in Figure 7).

## DISCUSSION

This study identified CD9 as a critical and indirectly druggable node in the neuroblastoma invasion-metastasis cascade. This is of particular interest since widespread and extensive metastatic burden causes most neuroblastoma-related deaths, mirroring the perception that activating invasion and metastasis belongs to the cancer hallmarks acquired during the multistep development of human tumors [26]. Suppressing neuroblastoma metastasis would, taken in this light, be a top priority to improve survival of high-risk disease with targeted therapeutic approaches.

Neuroblastoma originates from aberrant neural crest progenitor cells, which physiologically give rise both to sympathetic neurons and chromaffin cells of the adrenal glands [1, 2]. A review of the literature indicates that CD9 is expressed in early neural crest-derived embryonic rat sympathetic neurons and adrenal chromaffin cells. CD9 expression is also sustained throughout adulthood, localized to the surface of nerve cell bodies, axons and growth cones [27]. Yet, so far, no role for CD9 has been described in neuroblastoma pathogenesis. We detected low-level *CD9* expression in primary neuroblastomas that correlated with metastasized high-risk disease, low probability of patient survival and established clinical and molecular markers for unfavorable disease including *MYCN* amplification in the tumor. High-level *CD9* expression in primary neuroblastomas served as an independent favorable prognostic marker. Patients stratified into subgroups based on different *CD9* expression levels in the tumors also segregated known prognostic markers such as age at diagnosis (≤ or > 18 months), and INSS tumor stages 3 or 4 *versus* stages 1, 2 or 4S. The major strength of *CD9* tumor expression as a novel prognostic marker may be its potential for predicting disease progression in neuroblastoma patient subgroups.

Using functional assays, we demonstrated that CD9 suppresses neuroblastoma cell migration and invasion. This is in line with evidence from studies with ovarian [28] and bladder carcinoma cell lines [29] that low-level CD9 expression triggers tumor cell motility and invasiveness. On a molecular level, CD9 is known to inhibit integrin-mediated motility and associate with the platelet aggregation-inducting factor podoplanin, thereby preventing platelet aggregation and, consequently, a tumor-cell protective microenvironment (reviewed in [30]). We moved beyond *in vitro* assays for invasion and metastasis, to an *in vivo* model where human neuroblastoma cells explanted to the chicken chorioallantoic membraneinvade the chicken embryo bone marrow, thus, reflecting human neuroblastoma metastatic behavior. Enforced CD9 expression strongly inhibited the number of neuroblastoma cells metastasized to the chicken bone marrow demonstrating that CD9 acts to inhibit the invasion-metastasis cascade in an *in vivo* neuroblastoma model. These data indicate that pharmacological strategies to raise CD9 expression in the tumor could potentially prevent an increasing burden of metastatic colonization in high-risk neuroblastoma patients.

Here we identified GRHL1 as a transcriptional activator of the *CD9* gene and showed that MYCN and HDAC5 transcriptionally repress *CD9* in neuroblastoma. Our experiments with histone deacetylase inhibitors demonstrated the potential indirect druggability of *CD9.* Statistical models identified the synergistic effect of combining an HDAC inhibitor and the DNA methyltransferase inhibitor, 5′-azadeoxycytidine, supporting the observation that epigenetic events can be remodeled by pharmacological intervention. Previous investigations by us and others [31–35] have delineated and deciphered specific functions of single HDACs in neuroblastomas with defined molecular profiles. We showed that HDAC2 cooperates with MYCN to suppress apoptosis mediated by miR-183 [36], and that HDAC3 interacts with MYCN to transcriptionally repress the *GRHL1* transcription factor, which exerts tumor suppressive effects in neuroblastoma [19]. We demonstrated that HDAC8 inhibits neuroblastoma cell differentiation [37] and HDAC10 promotes autophagy-mediated neuroblastoma cell survival [38]. We detected elevated levels of HDAC11 expression in neuroblastomas and several carcinomas compared to corresponding healthy tissues, and showed that HDAC11 depletion was sufficient to cause apoptosis and inhibit metabolic activity in different cancer cell lines, representing major oncological indications for industrial drug development programs [39]. Beyond the previously described specific and non-redundant oncogenic functions of HDAC2, HDAC3, HDAC8, HDAC10 and HDAC11 in neuroblastoma pathophysiology, this study defines a role for HDAC5 in triggering the invasion-metastasis cascade in neuroblastoma.

As we are only at the beginning of understanding the potential therapeutic use of HDAC inhibition strategies and their combination within complex rationales utilizing several epigenetic-cancer therapies, further studies are required to define how to best construct drug strategies targeting epigenetic players such as HDACs and bromodomain and extra-terminal domain (BET) protein family members. Taken together, the present study (i) identifies low-level *CD9* expression as a novel prognostic marker in high-risk neuroblastoma patient subgroups, (ii) exposes *CD9* transcriptional activation by GRHL1 and its repression by MYCN and HDAC5, (iii) defines the potential of high-level CD9 expression to interrupt the neuroblastoma invasion-metastatic cascade and (iv) demonstrates the indirect druggability of CD9 by combined inhibition of HDAC5 and DNA methyltransferase inhibitors.

## MATERIALS AND METHODS

### Tumor samples and cell culture

Formalin-fixed, paraffin-embedded samples from 20 primary neuroblastomas (13 localized or stage 4S and 7 stage 4, 3 of which harboring *MYCN* amplifications) available from patients in the NB2004 trial (informed consent available) were selected for CD9 immunohistochemistry. Authenticated neuroblastoma cell lines and models were maintained under standard cell culture conditions. Details are supplied in Supplementary Materials and Methods.

### Quantitative reverse-transcriptase real-time PCR (qRT-PCR)

Total RNA was isolated from cell lines using the RNeasy Mini Kit (Qiagen). The Thermo Scientific First Strand cDNA Synthesis Kit was used to transcribe cDNAs for qRT-PCR analysis. Relative gene expression was measured in qRT-PCR using SYBR Green Dye (Eurogentec) and primers (Supplementary Table S1) on an ABI Prism 7500 thermal cycler (Perkin-Elmer Applied Biosystems), and normalized to the averaged expression of *ACTB* and *18S* rRNA or the averaged expression of *SDHA* and *HPRT1*, a gene pair that is consistently expressed in stage 4 and 4S neuroblastomas [40]. Data were analyzed using Applied Biosystems 7500 software v2.0.5, and changes in expression were calculated using the ΔΔC_t_ method [41].

### *In vitro* cell migration and invasion

Migration and invasion of cells was measured using the CIM-Plate 16 Boyden chamber system (ACEA Biosciences) and the xCELLigence Real-Time Cell Analyzer (ACEA Biosciences) [42, 43]. Boyden chambers were prepared for invasion assays by adding 160μl FCS-containing medium to the lower chamber and successively adding 50μl serum-free medium, 20μl Matrigel diluted 1:40 in serum-free medium followed by 4h at 37°C, 5% CO_2_ and 30μl serum-free medium above the Matrigel to the upper chamber. After 24h in serum-free medium, 6×10^4^ cells were seeded into the upper chamber, and migrating/invading cells were monitored by serial impedance measurements [42, 43]. Assays were performed in quadruplicate and repeated three times. Cell migration and invasion were calculated using RTCA Software version 2.0 (ACEA Biosciences).

### TaqMan^®^-based chicken embryo metastasis assay

Fertilized patho­gen-free leghorn eggs (Charles River) were incubated at 37°C in 60% humidity with rotation in a Marsh Incubator (Lyon Electric). The allontoic vein was localized at day 10, over which a window was cut in the shell using a miniaturized rotating electric saw (Dremel) after removing air from the natural air space under mild vacuum pressure. Single-cell suspensions of empty vector- or CD9 plasmid-transfected BE(2)-C cells were prepared in serum-free medium, 2×10^6^ cells inoculated onto each chorioallantoic membrane and the windows sealed with cellotape. Embryos were sacrificed on day 17 (*n* = 18 per study group), and bone marrow was isolated and snap frozen for preparation of genomic DNA. The TaqMan-based ALU assay takes advantage of the abundantly present *ALU* family of short interspersed repeated DNA elements in the human genome to identify human DNA [44]. PCR reactions were carried out as described [45] using 2x qRT-PCR MasterMix (Eurogentec) and primers for YB8-ALU-S68, YB8-ALU-AS244 and YB8-ALU-167 (Supplementary Table 1). A standard curve was generated from DNA samples from 10,000, 1000, 100, 10 and 0 BE(2)-C cells mixed with 1μg of chicken liver genomic DNA each to quantify the BE(2)-C cells metastasized to the chicken bone marrow.

### TH-MYCN neuroblastoma progression model

TH-MYCN^+/+^ mice [46] were sacrificed at day 7 (*n* = 4) and day 14 (*n* = 4) of life to harvest sympathetic ganglia containing hyperplastic neuroblast foci, and at week 6 of life to harvest advanced neuroblastic tumors (*n* = 4). Sympathetic ganglia were also dissected from TH-MYCN^−/−^ mice at day 7 (*n* = 4), day 14 (*n* = 4) and week 6 (*n* = 4) of life to assess gene expression changes during normal development. Total RNA was isolated using the miRNeasy Mini Kit, and samples profiled on Agilent SurePrint G3 Gene Expression Microarrays according to the manufacturers' protocols. Data were summarized and normalized with the vsn method [47] in the R statistical programming language [48] using the *limma* package [49]. Linear regression analysis was performed to evaluate differential temporal expression in ganglia from wildtype mice and ganglia and tumors from transgenic mice.

#### Subcutaneous BE(2)-C xenograft tumor model in CB17-SCID mice

BE(2)-C cells were transfected for 24h with LacZ- or CD9-vectors in culture, then 2x 10^6^ viable cells suspended in 200μl Matrigel (BD Biosciences) were subcutaneously implanted in the flanks of 6-week old female CB17-SCID mice (*n* = 12 per study group). Animals were sacrificed 13 days after grafting. Tumor size was measured daily with a caliper, and volume calculated by π/6(w1×w2×w2), where w1 equals largest tumor diameter, and w2 equals smallest tumor diameter. Experiments conformed to regulatory standards and were approved by the local ethics committee.

## Data analysis

*CD9* mRNA expression values were calculated using existing whole-genome expression profiles from 476 primary neuroblastomas [21], and validated using whole-genome expression profiles from 122 further neuroblastomas [22]. Kaplan-Meier curves were generated for the 476 neuroblastoma cohort using the R Survival package and compared using log-rank testing. The *CD9* probesets with the highest average signal were selected for analysis. Relapse, progression and death from disease were considered events for calculation of event-free survival. Both overall and event-free survival were calculated from the time of diagnosis to death, event or last examination. Optimal cut-offs for overall and event-free survival analyses were calculated using the R Maxstat package according to the method defined by Lausen and Schumacher [50] determining the expression value best separating outcome into two groups using a maximally selected two-sample log-rank statistic. Optimal cut-offs were 4774.47 (overall survival) and 5330.71 (event-free survival). Results were corrected for multiple testing. *CD9* expression between two patient subgroups were compared using the Mann-Whitney U test. Univariate and multivariate analyses were performed for overall and event-free survival using Cox proportional hazards regression models using SPSS (IBM, 20.0.0 release) or R (version 3.0.1). The R2 platform (http://r2.amc.nl) was used to generate Kaplan-Meier survival curves and box plots of *CD9* expression comparisons for the 122-neuroblastoma cohort [22]. *CD9* gene methylation status was derived from existing methyl-CpG-binding domain sequencing data from 60 internationally collected primary neuroblastoma samples [24, 25]. Single values from cell culture experiments were compared using the one-sample *t*-test in GraphPad Prism version 5.0 (GraphPad Software Inc., La Jolla, CA). *CD9* expression in cell groups transfected with either of two negative controls or either of two HDAC-specific siRNAs were compared using a mixed linear model with fixed-factor treatment and random intercept for each siRNA using SAS PROC MIXED, Version 9.2 (SAS Institute Inc., Cary, NC, USA). Delta-Ct values were evaluated by a two-factorial mixed linear model with interaction and random effect for each experimental run to assess potential synergistic effects of combined HDAC inhibitor/5-aza-cytidine treatment on CD9 expression. *P* values below 0.05 were considered significant.

## SUPPLEMENTARY MATERIAL TABLES AND FIGURES


