# Supplementary material for: MYCN and HDAC5 transcriptionally repress CD9 to trigger invasion and metastasis in neuroblastoma
Source: Oncotarget. 2016 Aug 27;7(41):66344–59. doi: 10.18632/oncotarget.11662 (PMC5341807; doi:10.18632/oncotarget.11662)
Supplement: Supplementary file 1 [file oncotarget-07-66344-s001.pdf]

MYCN and HDAC5 transcriptionally repress *CD9* to trigger invasion and metastasis in neuroblastoma

Supplementary Material

Table of Contents

Supplementary Materials and Methods	starting p.2
Supplementary Tables	starting p.6
Supplementary Figures	starting p.9
Legends to Supplementary Figures	starting p.9
Supplementary References	starting p.14

SUPPLEMENTARY MATERIALS AND METHODS

Cell culture

The BE(2)-C cell line was obtained from ECACC (Salisbury, UK) and the IMR-32, Kelly and SH-SY5Y cell lines from the DSMZ (Braunschweig, Germany). SH-EP and the synthetic MYCN-inducible SH-EP Tet-21/N cell model [1] were kindly provided by M. Schwab. BE(2)-C, IMR-32, Kelly and SH-SY5Y were cultured in DMEM (Lonza) supplemented with 10% FCS (Sigma-Aldrich) and 1% NEAA (Lonza) at 37° C and 5% CO_2. SH-EP was cultured in RPMI 1640 Medium (Lonza) supplemented with 10% FCS and 1% NEAA at 37° C and 5% CO_2. SH-EP Tet-21/N cells were cultured in RPMI 1640 medium with HEPES (Lonza) supplemented with 10% FCS, 1% NEAA, 200µg/ml G418 (Merck Millipore) and 100µg/ml hygromycin B (Sigma-Aldrich), and treated with 1µg/ml tetracycline (AppliChem) to suppress synthetic *MYCN* expression [1]. The stable CD9-inducible model was generated by electroporating pcDNA™6/TR (Invitrogen) and pT-Rex™-DEST30-CD9 (RefSeq NM_001769; Invitrogen) into SH-EP cells, with control cells containing pcDNA™6/TR and pT-Rex™-DEST30. Selection medium contained 7.5µg/ml blasticidin and 0.5mg/ml geneticin, and single-cell clones were obtained by limited dilution. CD9 expression was induced by 1µg tetracycline/ml medium, and the clone with the strongest CD9 induction was identified by western blotting and flow cytometry (Supplementary Figure S3A-B). Cell lines were monitored for infections by high-throughput multiplex cell contamination testing [2]. Cell line authenticity was validated by high-throughput SNP-based assays [3].

Reagents

Stock solutions of 5-Aza-2'-deoxycytidine (10 mM; Sigma-Aldrich), panobinostat (1 mM; Selleck Chemicals), vorinostat (1 mM; Chemos), compound 2 (250 mM; Merck Millipore), tubacin (1 mM; Selleck Chemicals) and bufexamac (100 mM; Sigma-Aldrich) were prepared

in DMSO [4]. Actinomycin D (stock solution 5 mg/ml in DMSO; Sigma-Aldrich) was added at a final concentration of 1µg/ml to the medium [4].

Transfection of siRNAs, shRNA and DNA plasmids

Cells were transiently transfected with 25 nM siRNA (Supplementary Table S2) using the HiPerFect (Qiagen) method according to the manufacturer's directions. DNA and shRNA plasmids were transiently transfected using the Effectene (Qiagen) method according to the manufacturer's directions. The *MYCN* shRNA plasmid was kindly provided by F. Westermann (Neuroblastoma Genetics, DKFZ, Heidelberg, Germany), and the pTER(+) vector [5] containing a scrambled shRNA sequence served as control. Applying the GATEWAY technology (Invitrogen, Darmstadt, Germany), the *CD9* sequence was cloned from the gateway vector IOH40154 (Invitrogen) into the destination vector pT-REX-DEST30 (Invitrogen) (Supplementary Fig. S3C-D). The empty pT-REX-DEST30 vector and the LacZ expression vector, pT-REX/GW30/LacZ (Invitrogen), served as controls. The HDAC5-FLAG pcDNA3.1 plasmid [6] was kindly provided by J. Backs (Cardiac Epigenetics, University Hospital Heidelberg, Germany), for which the pcDNA3.1 vector served as a control. To perform anti-FLAG-GRHL1 ChIP experiments, the *GRHL1* sequence was cloned from the destination vector pT-REX-DEST30 [4] into the destination vector pT-REx™-DEST31 applying the GATEWAY technology (Invitrogen).

Chromatin immunoprecipitation and qPCR

Cells were lysed for ChIP in buffer containing 50 mM Tris-HCl, 1% SDS, 10 mM EDTA and Complete® protease inhibitor cocktail (Roche, Mannheim, Germany), then sonicated to obtain chromatin fragments of 100 to 300 base pairs. Anti-MYCN and anti-pan-acetylated histone H4 ChIPs were performed according to the ChIP Assay Kit protocol (Merck Millipore). The following antibodies were used: mouse monoclonal anti-MYCN (clone B8.4.B; Santa Cruz Biotechnology), rabbit polyclonal anti-pan-acetylated histone H4 (Merck Milli-

pore) and mouse or rabbit IgG (Santa Cruz Biotechnology) as negative controls. In anti-FLAG-GRHL1 and anti-HDAC5-FLAG ChIPs, immunocomplexes were captured with anti-rabbit IgG TrueBlot beads (eBioscience, Frankfurt, Germany) or with anti-FLAG M2-conjugated agarose (Sigma-Aldrich) by incubation at 4 °C overnight on a rotatory mixer. Primers for qRT-PCR amplification of the *CD9* promoter region were: 5'-TTTTTAAAAGTGCAGCCGGAGA-3' (forward), 5'-CATCTGTATCCAGCGCCAGG-3' (reverse).

Western blotting, immunohistochemistry and flow cytometry

Cells were lysed for western blotting in buffer containing 20 mM Tris-HCl, 7 M urea, 0.01% Triton X-100, 100 mM DTT, 40 mM $MgCl_2$ and Complete® protease inhibitor cocktail [7]. The following antibodies were used: mouse monoclonal anti-β-actin (clone AC-15, Sigma-Aldrich), mouse monoclonal anti-CD9 (clone C-4, Santa Cruz Biotechnology), mouse monoclonal anti-GAPDH (clone 6C5, Merck Millipore), rabbit polyclonal anti-GRHL1 (Sigma-Aldrich), rabbit polyclonal anti-HDAC5 (Cell Signaling), rabbit polyclonal anti-histone H3 (Cell Signaling) and mouse monoclonal anti-MYCN (clone B8.4.B; Santa Cruz Biotechnology). Band density was analyzed using ImageJ 1.47p software [8] on western blots, and results were normalized to the respective loading controls. The mouse monoclonal anti-CD9 antibody (clone C-4, Santa Cruz Biotechnology; [9]) was also used for immunohistochemistry on 20 formalin-fixed, paraffin-embedded tumor sections. In brief, tumor sections were freshly cut to 4 µm and dried at 80° C for 15 min. CD9-specific immunohistochemistry was performed on an automated immunostainer (Ventana BenchMark XT, Ventana Medical Systems, Tucson, AZ) using the OptiView DAB IHC Detection Kit and standard reagents provided by Ventana. After completion of the immunohistochemical staining, sections were counterstained with hematoxylin to visualize the nuclei in the tumor sections. CD9 protein expression in tumor samples was semi-quantitatively analyzed by counting the percentage of CD9-positive tumor cells and by scoring the staining intensity [10]. Scoring was done in consensus

of two investigators. Digitized images were generated using a Leica DMRBE microscope, SPOT CCD camera and Software SPOT2.1.2. For anti-CD9 flow cytometry, cells were incubated with 50 µl primary antibody (clone C-4, Santa Cruz Biotechnology) at pretested concentration (30 min, 4 °C), washed three times, incubated with dye-labeled secondary antibody (30 min, 4 °C) and washed again. Cells were analyzed in a FACScan using the Cell Quest analysis program (BD, Heidelberg, Germany).

Proliferation assay

Cell viability and number were measured with the VI-CELL Cell Viability Analyzer (Beckman, Krefeld, Germany) based on the trypan blue-exclusion method [4].

Supplementary Table S1. Primers used in qRT-PCR [4, 11].

Gene	Forward primer (5’-3’)	Reverse primer (5’-3’)	Order ID#*
ACTB	GCATCCCCCAAAGTTCACAA	AGGACTGGGCCATTC TCCTT	
CD9	GGACGTACTCGAAACCTTCACC	GCGGATAGCACAGCA CAAGA	
GRHL1			QT00067025
HDAC1	TGACGAGTCCTATGAGGCCATT	CCGCACTAGGCTGGAACATC	
HDAC2	TGTGAGATTCCCAATGAGTTGC	GGTAACATGCGCAAATTTTCAA	
HDAC3	CCTCACTGACCGGGTCATG	ACCTGTGCCAGGGAAGAAGTAA	
HDAC4	GAGGTTGAGCGTGAGCAAGAT	TAGCGGTGGAGGGACATGTAC	
HDAC5	GTCTCGGCTCTGCTCAGTGTAGA	GGCCACTGCGTTGATGTTG	
HDAC6	CAAGGAACACAGTTCACCTTCG	GTTCCAAGGCACATTGATGGTA	
HDAC7	AGGACAAGAGCAAGCGAAGTG	TTCAGAATCACCTCCGCTAGCT	
HDAC8	CCAAGAGGGCGATGATGATC	GTGGCTGGGCAGTCATAACC	
HDAC9	AGTGTGAGACGCAGA CGCTTAG	TTTGCTGTCGCATTTGTTCTTT	
HDAC10	ATCTCTTTGAGGATGACCCCAG	ACTGCGTCTGCATCTGACTCTC	
HDAC11	CAATGGGCATGAGCGAGAC	TGTGGCGGTTGTAGACATCC	
HPRT1	TGACACTGGCAAAACAATGCA	GGTCCTTTTCACCAGCAAGCT	
SDHA	TGGGAACAAGAGGGCATCTG	CCACCACTGCATCAAATTCATG	
18-S			QT00199367

* Qiagen

Supplementary Table S2. Purchasing information for siRNAs [4, 11].

Gene	siRNA abbreviation	Company	Order ID#
GRHL1	si-1	Qiagen	SI04251331
	si-2	Qiagen	SI04346545
HDAC1	si-1	Qiagen	SI00070609
	si-2	Qiagen	SI00070623
HDAC2	si-1	Invitrogen	120209
	si-2	Invitrogen	120210
HDAC3	si-1	Qiagen	SI00057337
	si-2	Qiagen	SI03057901
HDAC4	si-1	Qiagen	SI03082282
	si-2	Invitrogen	107926
HDAC5	si-1	Qiagen	SI00077714
	si-2	Qiagen	SI00077735
HDAC6	si-1	Qiagen	SI00084000
	si-2	Qiagen	SI04438490
HDAC7	si-1	Qiagen	SI02777726
	si-2	Qiagen	SI04439715
HDAC8	si-1	Qiagen	SI00122080
	si-2	Qiagen	SI03049382
HDAC9	si-1	Qiagen	SI00148372
	si-2	Qiagen	SI00148393
HDAC11	si-1	Qiagen	SI03039085
	si-2	Qiagen	SI03084158
	siNC-1	Qiagen	1027281
	siNC-2	Thermo Scientific	D-001220-01-20

Supplementary Table S3. High-level *CD9* mRNA expression in neuroblastomas serves as an independent prognostic marker for favorable patient outcome.

Univariate Cox regression

		**Event-free survival**		**Overall survival**	
**Factor**	***n***	**Hazard Ratio (95% CI)**	***P***	**Hazard Ratio (95% CI)**	***P***
CD9	476	0.14 (0.08; 0.24)	< 0.001	0.04 (0.02; 0.09)	< 0.001
INSS stage	476		< 0.001		< 0.001
I, II, III (ref.)	267		-		-
IV	148	3.96 (2.83; 5.54)	-	7.78 (4.83; 12.52)	-
IVs	61	1.18 (0.66; 2.13)	-	1.02 (0.39; 2.70)	-
MYCN	471		< 0.001		< 0.001
Single-copy (ref.)	405		-		-
Amplified	66	3.68 (2.60; 5.20)	-	7.88 (5.29;11.74)	-
Age at diagnosis	476		< 0.001		< 0.001
≤ 18 months (ref.)	309		-		-
> 18 months	167	3.49 (2.54; 4.80)	-	9.38 (5.75; 15.31)	-

Multivariate Cox regression

		**Event-free survival**		**Overall survival**	
**Start model**	***n***	**HR (95% CI)**	***P***	**HR (95% CI)**	***P***
CD9	471	0.40 (0.23; 0.72)	0.001	0.36 (0.17; 0.79)	0.005
INSS stage			0.001		< 0.001
I, II, III (ref.)			-		-
IV		2.06 (1.39; 3.05)	-	2.84 (1.68, 4.81)	-
IVs		1.50 (0.81; 2.76)	-	1.84 (0.66; 5.18)	-
MYCN			0.049		< 0.001
Single-copy (ref.)			-		-
Amplified		1.51 (1.01; 2.28)	-	2.45 (1.53; 3.93)	-
Age at diagnosis			< 0.001		< 0.001
≤ 18 months (ref.)			-		-
> 18 months		2.13 (1.41; 3.20)	-	4.14 (2.24; 7.64)	-

NOTE: *CD9* mRNA expression values were derived from existing whole-genome expression profiles of 476 neuroblastoma samples [12]. Univariate and multivariate analyses were performed for EFS and OS using Cox's proportional hazards regression models. For this purpose, CD9 was scaled with factor 10^{-4} to minimize the wide range of the data. The factors *CD9* (scaled and continuous), INSS stage (I, II, III (ref.) *versus* IV *versus* IVS), *MYCN* (single-copy (ref.) *versus* amplified) and age at diagnosis (≤ 18 months (ref.) *versus* > 18 months) were fitted into a backward selection. The criterion for inclusion was a likelihood-ratio test p-value less than 0.05 and for exclusion more than 0.10. Calculations were performed in IBM SPSS package (Release 20.0.0) or packages from R version 3.0.1.

Supplementary Figure S1. Related to Figure 2.

Supplementary Figure S2. Related to Figure 2.

Supplementary Figure S3. Related to Figure 5.

Supplementary Figure S4. Related to Figure 6.

Supplementary Figure S5. Related to Figure 6.

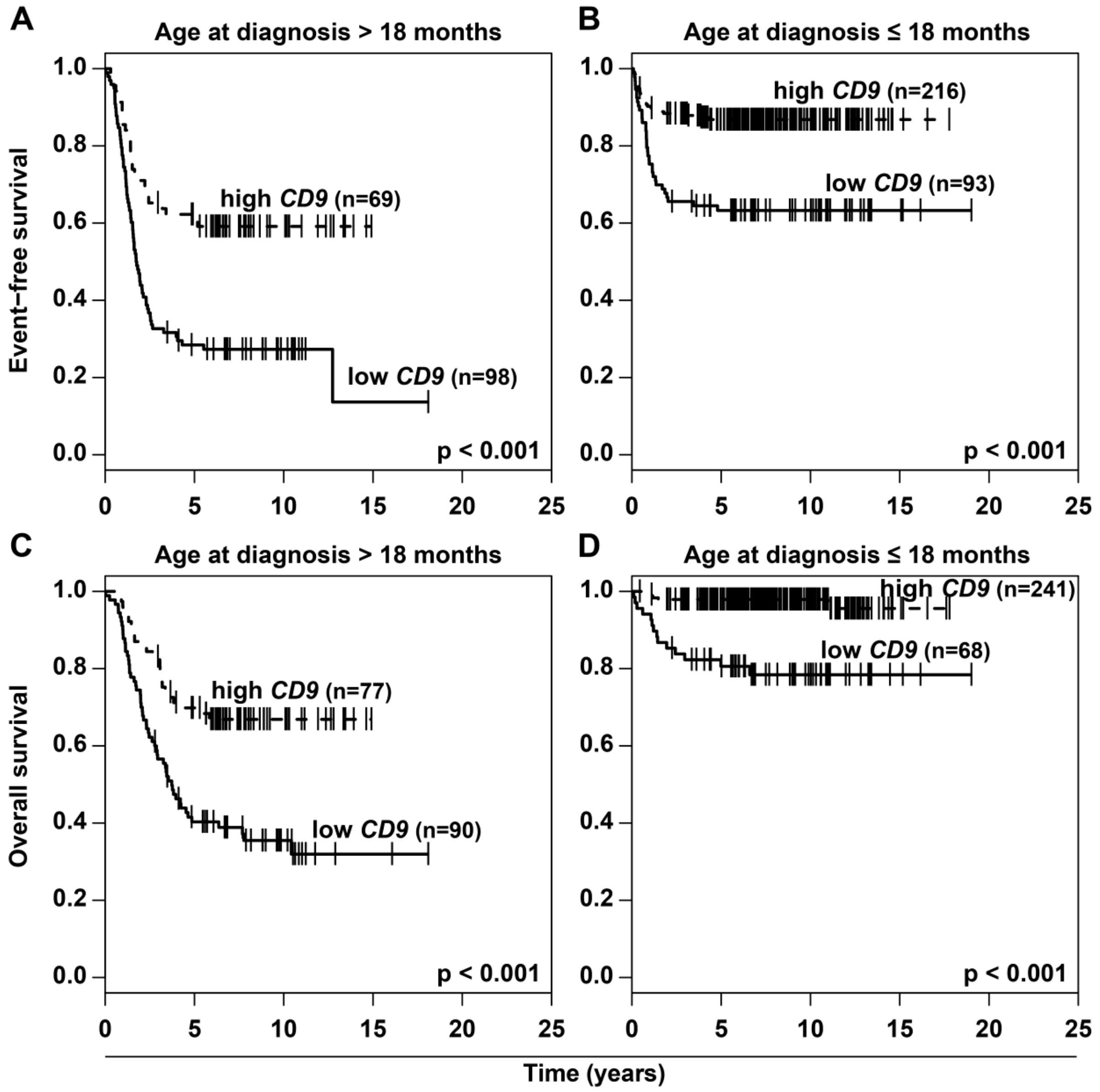

Supplementary Figure S1. High-level *CD9* expression in neuroblastomas predicts favorable patient survival irregardless of age at initial diagnosis. Kaplan-Meier analyses of event-free (A-B) and overall survival (C-D).

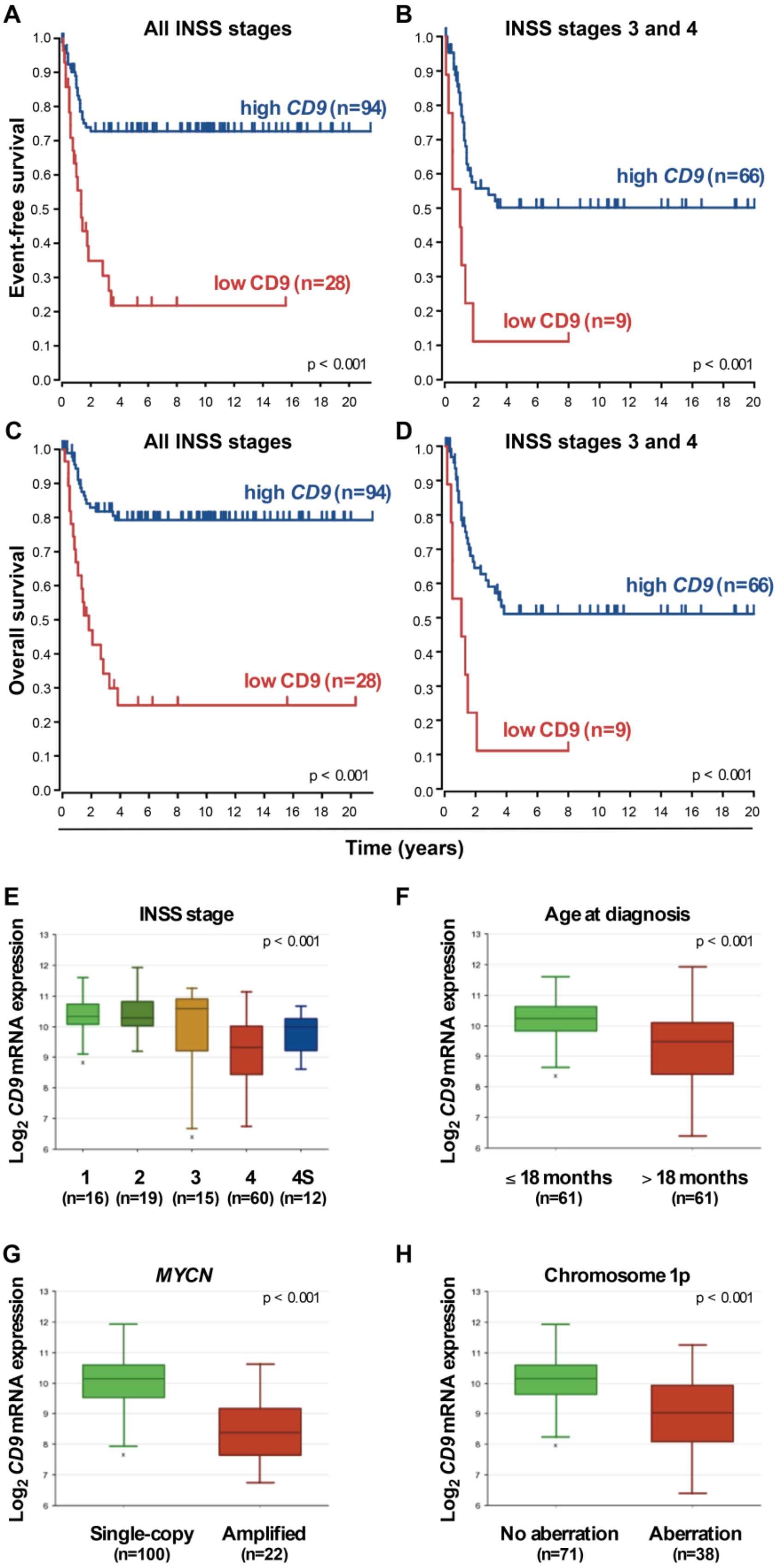

Supplementary Figure S2. High-level *CD9* expression in neuroblastomas predicts favorable patient survival in the second independent 122-tumor cohort [13] and correlates with favora-

ble clinical and prognostic markers. Kaplan-Meier analysis shows how *CD9* tumor expression correlated with event-free (A-B) and overall survival (C-D) irregardless of INSS stage (A, C) and in INSS stages 3 and 4 (B, D). Boxplots compared *CD9* expression in individual INSS stages (E), tumors from patients diagnosed above or under 18 months of age (F), tumors lacking or harboring *MYCN* amplifications (G) and tumors either lacking or harboring 1p aberrations (H). All analyses were performed in the R2 microarray analysis and visualization platform (http://r2.amc.nl), and the resulting survival curves and *P* values (log-rank test) were downloaded. Cut-off values for separating tumors with high and low levels of *CD9* expression were also determined using the R2 platform tools. INSS, International Neuroblastoma Staging System.

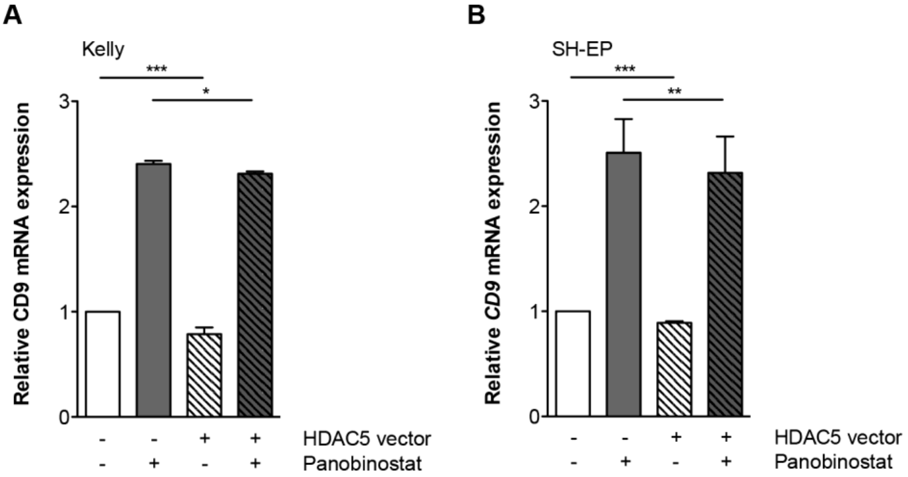

Supplementary Figure S3. HDAC5 negatively regulates endogenous *CD9* expression and counteracts *CD9* induction by panobinostat treatment. *CD9* mRNA expression (assessed by qRT-PCR) in Kelly (A) and SH-EP cells (B) transfected with HDAC5 or empty vector for 72h and treated with 15 nM panobinostat or solvent control for 48h. *$P < 0.05$; **$P \leq 0.01$; ***$P \leq 0.001$.

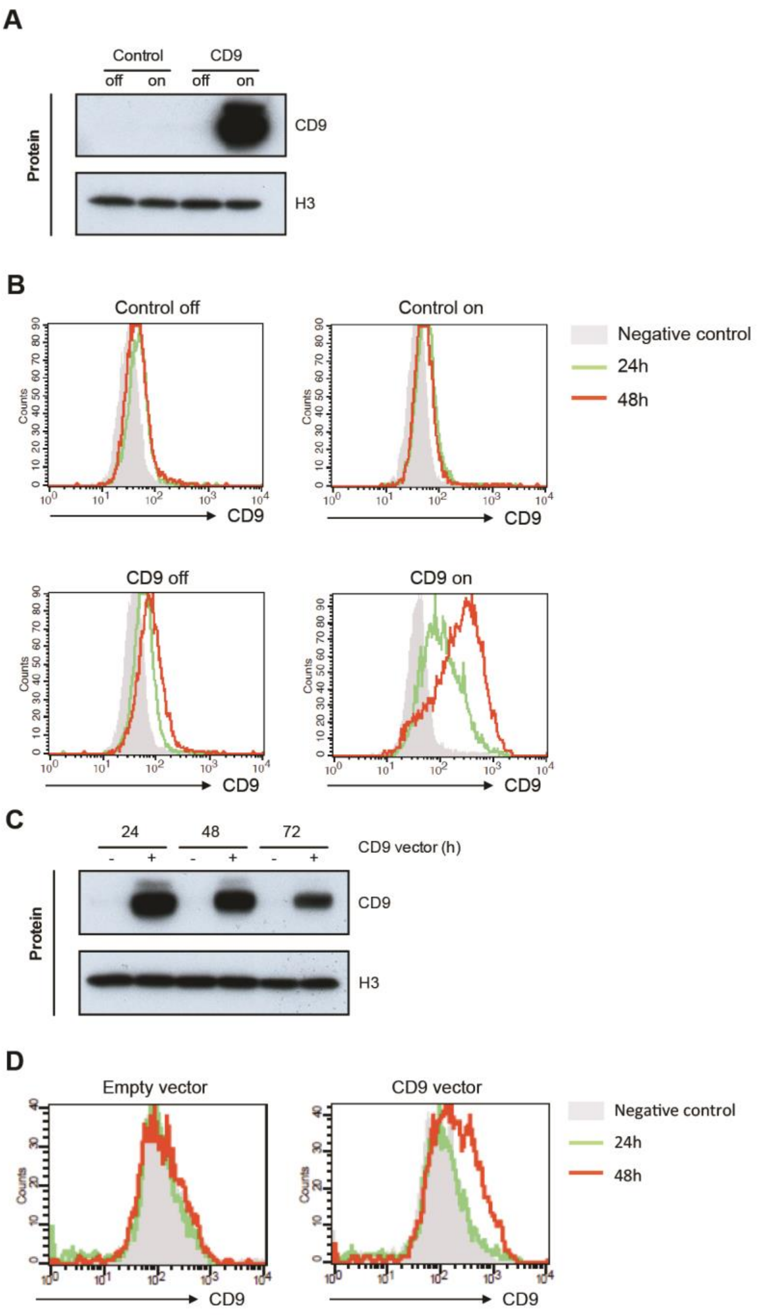

Supplementary Figure S4. Characterization of cell models for functional analysis. A-B, CD9 induction in the stable CD9-inducible SH-EP model. CD9 expression was analyzed at the protein level by western blotting (A) and flow cytometry (B) 24-48 hours after treating the stable CD9-inducible and control SH-EP clone with 1µg/ml tetracycline. C-D, enforced CD9 expression in BE(2)-C cells. CD9 expression was analyzed at the protein level by western blotting (C) and flow cytometry (D) 24-72 hours after transient transfection with empty- or

CD9 vector. Histone H3 served as loading control. Representative blots and images are shown.

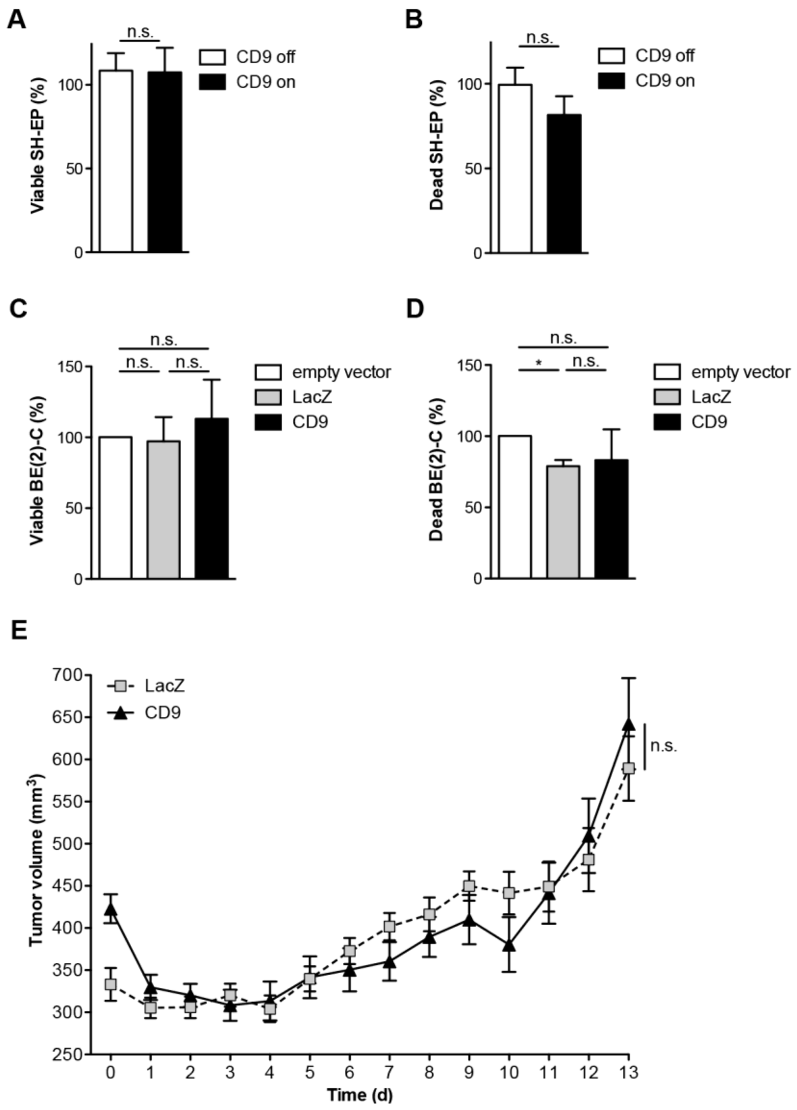

Supplementary Figure S5. Effect of CD9 expression in neuroblastoma cells on proliferation, viability and subcutaneous xenograft growth in mice. A-B, proliferation assay indicating the number of viable (A) and dead SH-EP cells (B) 72 hours after induction of CD9 (mean ± SD, n = 5). C-D, proliferation assay indicating the number of viable (C) and dead BE(2)-C (D) 72h after transfection with empty-, LacZ-, or CD9 vector (mean ± SD, n = 5). E, BE(2)-C cells were transiently transfected with empty-, LacZ-, or CD9 vector 24h before xenografting into CB17-SCID mice. Shown is the time-course of BE(2)-C xenograft growth from the day of implantation (day 0) to day 13 (n=12 per study group). n.s., not significant. $^{*}P < 0.05$.

SUPPLEMENTARY REFERENCES

1. Lutz W, Stohr M, Schurmann J, Wenzel A, Lohr A and Schwab M. Conditional expression of N-myc in human neuroblastoma cells increases expression of alpha-prothymosin and ornithine decarboxylase and accelerates progression into S-phase early after mitogenic stimulation of quiescent cells. Oncogene 1996; 13(4):803-812.
2. Schmitt M and Pawlita M. High-throughput detection and multiplex identification of cell contaminations. Nucleic Acids Res 2009; 37(18):e119.
3. Castro F, Dirks WG, Fahnrich S, Hotz-Wagenblatt A, Pawlita M and Schmitt M. High-throughput SNP-based authentication of human cell lines. Inter J Cancer 2013; 132(2):308-314.
4. Fabian J, Lodrini M, Oehme I, Schier MC, Thole TM, Hielscher T, Kopp-Schneider A, Opitz L, Capper D, von Deimling A, Wiegand I, Milde T, Mahlknecht U, Westermann F, Popanda O, Roels F, et al. GRHL1 acts as tumor suppressor in neuroblastoma and is negatively regulated by MYCN and HDAC3. Cancer Res 2014; 74(9):2604-2616.
5. van de Wetering M, Oving I, Muncan V, Pon Fong MT, Brantjes H, van Leenen D, Holstege FC, Brummelkamp TR, Agami R and Clevers H. Specific inhibition of gene expression using a stably integrated, inducible small-interfering-RNA vector. EMBO reports 2003; 4(6):609-615.
6. Backs J, Backs T, Bezprozvannaya S, McKinsey TA and Olson EN. Histone deacetylase 5 acquires calcium/calmodulin-dependent kinase II responsiveness by oligomerization with histone deacetylase 4. Mol Cellular Biol 2008; 28(10):3437-3445.
7. Deubzer HE, Ehemann V, Westermann F, Heinrich R, Mechtersheimer G, Kulozik AE, Schwab M and Witt O. Histone deacetylase inhibitor Helminthosporium carbonum (HC)-toxin suppresses the malignant phenotype of neuroblastoma cells. Inter J Cancer. 2008; 122(8):1891-1900.
8. Schneider CA, Rasband WS and Eliceiri KW. NIH Image to ImageJ: 25 years of image analysis. Nat Methods 2012; 9(7):671-675.
9. Huan J, Gao Y, Xu J, Sheng W, Zhu W, Zhang S, Cao J, Ji J, Zhang L and Tian Y. Overexpression of CD9 correlates with tumor stage and lymph node metastasis in esophageal squamous cell carcinoma. Int J Clin Exp Pathol 2015; 8(3):30 54-3061.
10. Remmele W and Stegner HE. Recommendation for uniform definition of an immunoreactive score (IRS) for immunohistochemical estrogen receptor detection (ER-ICA) in breast cancer tissue. Der Pathologe. 1987; 8(3):138-140.
11. Lodrini M, Oehme I, Schroeder C, Milde T, Schier MC, Kopp-Schneider A, Schulte JH, Fischer M, De Preter K, Pattyn F, Castoldi M, Muckenthaler MU, Kulozik AE, Westermann F, Witt O and Deubzer HE. MYCN and HDAC2 cooperate to repress miR-183 signaling in neuroblastoma. Nucleic Acids Res 2013; 41(12):6018-6033.
12. Oberthuer A, Juraeva D, Li L, Kahlert Y, Westermann F, Eils R, Berthold F, Shi L, Wolfinger RD, Fischer M and Brors B. Comparison of performance of one-color and two-color gene-expression analyses in predicting clinical endpoints of neuroblastoma patients. Pharmacogenomics J 2010; 10(4):258-266.
13. Molenaar JJ, Koster J, Zwijnenburg DA, van Sluis P, Valentijn LJ, van der Ploeg I, Hamdi M, van Nes J, Westerman BA, van Arkel J, Ebus ME, Haneveld F, Lakeman A, Schild L, Molenaar P, Stroeken P, et al. Sequencing of neuroblastoma identifies chromothripsis and defects in neuritogenesis genes. Nature 2012; 483(7391):589-593.
